# Bronchiectasis Development in a Male Patient With a History of Tuberculosis: A Case Report

**DOI:** 10.7759/cureus.66866

**Published:** 2024-08-14

**Authors:** Ankit Rangari, Babaji Ghewade, Gauri Gajabe

**Affiliations:** 1 Respiratory Medicine, Jawaharlal Nehru Medical College, Datta Meghe Institute of Higher Education and Research, Wardha, IND; 2 Clinical Embryology, Jawaharlal Nehru Medical College, Datta Meghe Institute of Higher Education and Research, Wardha, IND

**Keywords:** mucociliary clearance, high resolution computed tomography (hrct), bronchiectasis, pulmonary tuberculosis, pulmonary function test (pft)

## Abstract

Bronchiectasis is a chronic respiratory disease characterized by a syndrome of productive cough and recurrent respiratory infections due to permanent dilatation of the bronchi. In this case, we discuss a 32-year-old male patient with a history of tuberculosis (TB) from a rural area of Wardha, Maharashtra. The case discusses the diagnostic modalities confirming the diagnosis, sputum investigations, and imaging studies like chest X-ray, high-resolution computed tomography (HRCT), pulmonary function test (PFT), and bronchoscopy. This case underscores the importance of early recognition and management of bronchiectasis in patients with a history of pulmonary TB. Chronic inflammation and necrosis from the initial TB infection likely contributed to impaired mucociliary clearance and bronchial dilation, creating a conducive environment for bacterial colonization and recurrent infections. This case highlights the need for long-term follow-up and potential interventions to manage chronic respiratory symptoms in post-TB patients.

## Introduction

Bronchiectasis is a progressive chronic respiratory disease of the bronchial tree characterized by irreversible dilatation and destructive changes in the walls of the bronchi. Pathological expansion of airways then occurs because of the vicious cycle of infection and inflammation, mucociliary clearance impairment, chronic cough, sputum production, and frequent respiratory infections [1]. Although the underlying causes of bronchiectasis can be pretty varied, with cystic fibrosis among the most common congenital conditions, recurrent infections like bacterial pneumonia, or autoimmune diseases, such as rheumatoid arthritis, post-infectious bronchiectasis still is the most prevalent etiology in countries with high prevalence of tuberculosis (TB) [2]. The condition affects not only the quality of life but also the progressive nature of symptoms and associated complications, which come as a challenge. In this study, the pathophysiology and clinical manifestations of bronchiectasis are identified so that therapeutic strategies can be suitably chosen and used in a way that optimizes patient care and improves long-term outcomes [3].

## Case presentation

A 32-year-old male patient from a rural area came to the outpatient department of respiratory medicine with a history of pulmonary TB one year back and took anti-tubercular medicines Isoniazid 75 mg, Rifampicin 150 mg, Pyrazinamide 400 mg, and Ethambutol 275 mg for six months. The patient came with a complaint of chest pain and swelling over the neck for one year. He also complains of breathlessness on exertion and cough with whitish expectoration for one year. He gave a history of tobacco consumption for eight years but had quit five years ago, along with exposure to biomass. On examination, blood pressure was 110/70 mmHg, pulse rate was 80 beats per minute, respiratory rate was 16 breaths per minute, and oxygen saturation was 98% on room air, all within normal range. An examination of the chest reveals that bilateral bronchial breath sounds were heard, suggesting respiratory issues. The cardiovascular and central nervous system examinations were normal.

Diagnostic workup

**Figure 1:**
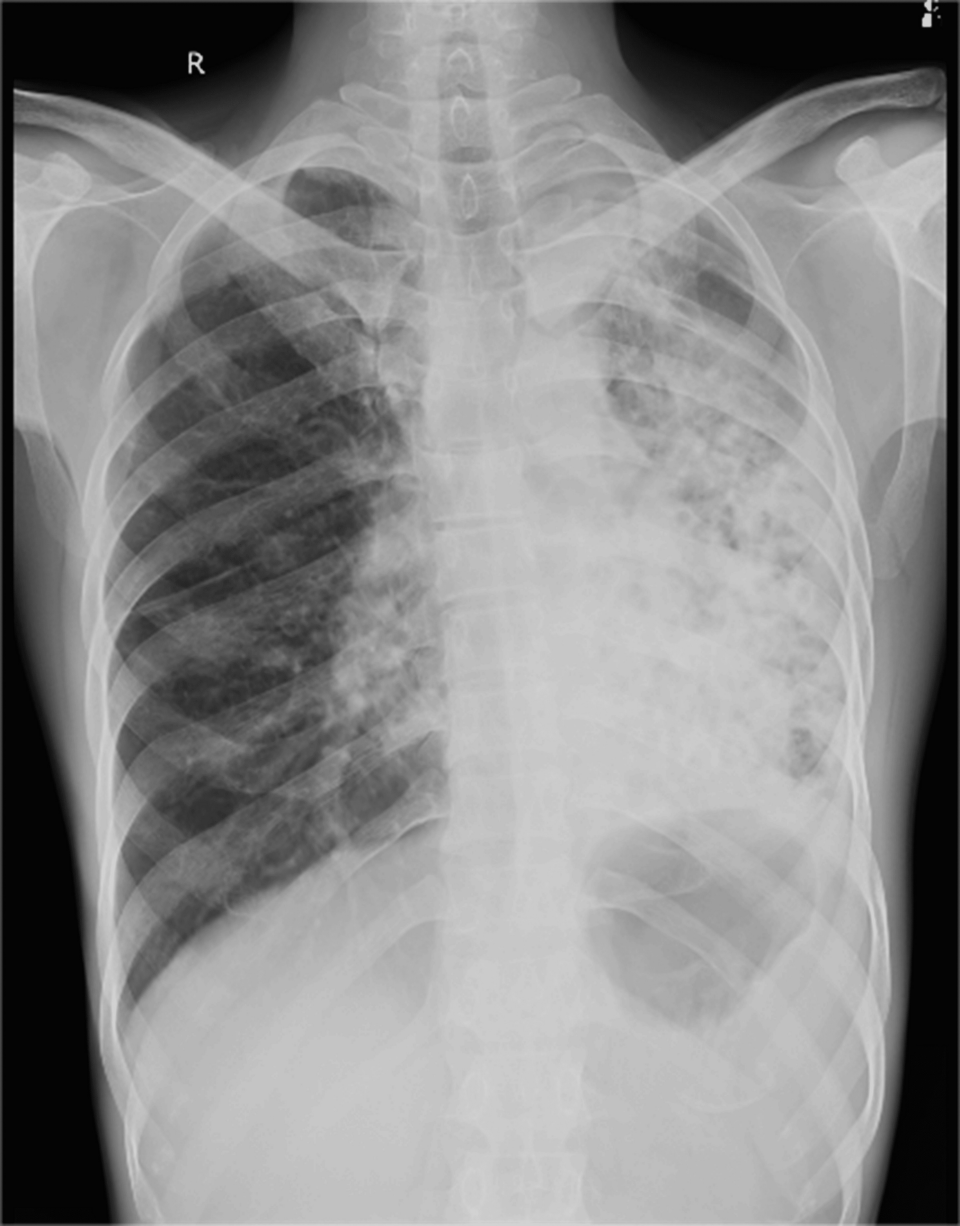
Chest X-ray suggestive of fibrocavitary lesions on the left side

**Figure 2:**
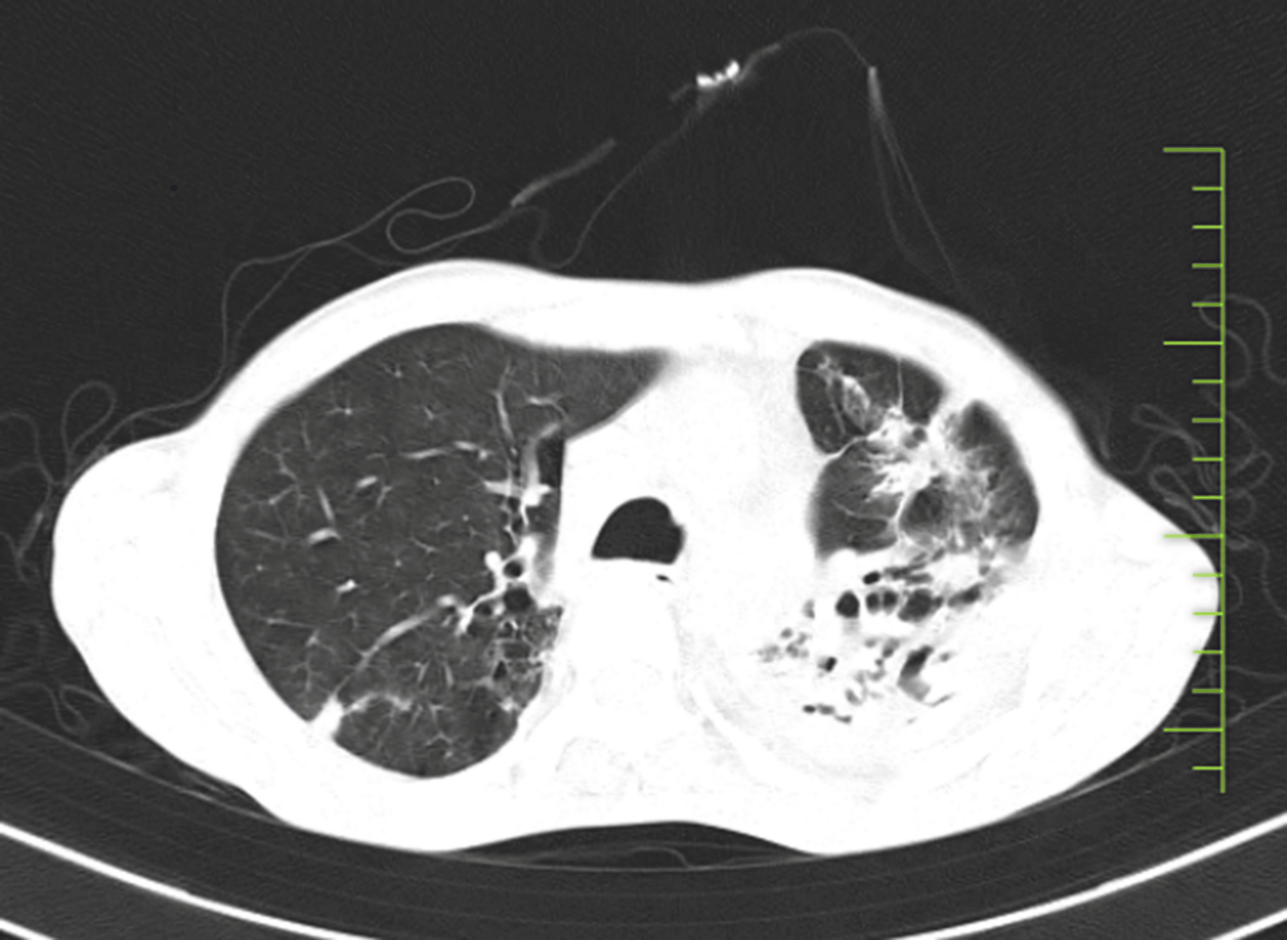
High-resolution computed tomography (HRCT) thorax suggestive of old tuberculosis with active infective etiology

**Figure 3:**
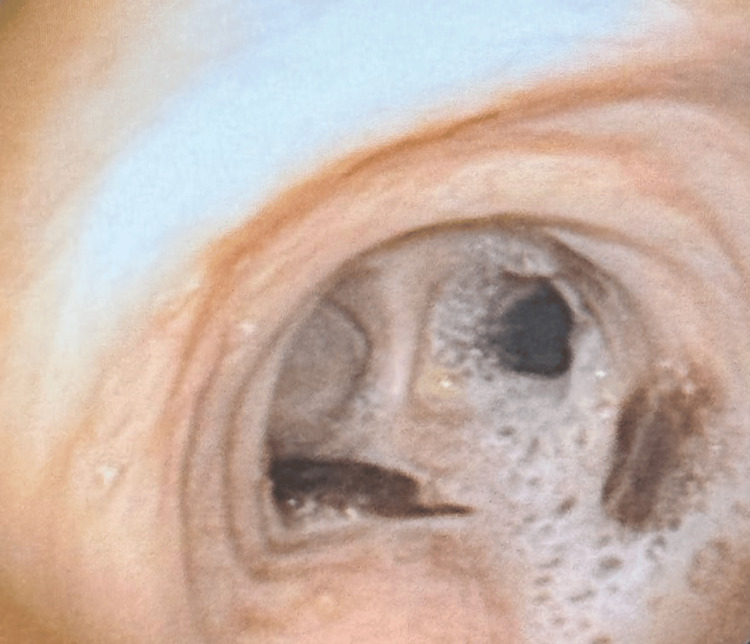
Bronchoscopic image showing obstruction and mucus with blood buildup in the bronchus

On admission, a chest X-ray was done, which was suggestive of fibrocavitary lesions on the left side, as shown in Figure 1. A sputum acid-fast bacilli (AFB) test was done to detect previous TB infection, but the result was negative. A routine blood investigation was advised, which revealed elevated blood parameters, as shown in Table 1. The pulmonary function test shows significant improvement post-bronchodilator. Forced vital capacity (FVC) increased from 1.45 L (48% predicted) to 2.60 L (87% predicted), indicating a 79% improvement. Forced expiratory volume in the first second (FEV_1_) improved from 0.61 L (26% predicted) to 1.13 L (49% predicted), a 5% increase. FEV_1_/FVC ratio slightly increased from 42.07% to 43.46%, indicating mild improvement in airway obstruction, as shown in Table 2. Further, a high-resolution computed tomography (HRCT) thorax was done, which shows cystic and varicoid bronchiectatic changes with fibrosis noted in the left lung, predominantly in the lower lobe and right middle lobe, with loss of left lung volume and compensatory hyperinflation of right lung-old TB, as shown in (Figure 2). Later on, a bronchoscopy procedure was advised in which we visualized bloody thick mucoid secretions present in both the lung lobes (Figure 3). Upon collection of the patient's bronchoalveolar lavage (BAL) specimen, it was sent to the laboratory. The test results indicate that AFB was not seen, and the Truenat test for TB was negative.

**Table 1:** Routine blood investigations of patient ALT, alanine aminotransferase; APTT, activated partial thromboplastin time; AST, aspartate aminotransferase; Hb, hemoglobin; INR, international normalized ratio; RBC, red blood cell; SGOT, serum glutamic-oxaloacetic transaminase; SGPT, serum glutamic-pyruvic transaminase; WBC, white blood cell

Investigations	Result	Normal values
Hb	10	Male: 14-18 g/dL; female: 12-16 g/dL
Total RBC count	3.52	Male: 4.7-6.1 million cells/mcL; female: 4.2-5.4 million cells/mcL
Total WBC count	5,200	4,500-11,000 cells/mcL
Total platelet count	4.44	150,000-450,000 platelets/mcL
APTT - control	29.5	25-35 seconds
APTT - patient	33.8	25-35 seconds
Prothrombin time - control	11.9	11-13.5 seconds
Prothrombin time - patient	18.4	11-13.5 seconds
INR	1.58	0.8-1.2 (for patients not on anticoagulant therapy)
Calcium	7.9	8.6-10.2 mg/dL
Urea	7	7-20 mg/dL
Creatinine	0.5	Male: 0.74-1.35 mg/dL; female: 0.59-1.04 mg/dL
Sodium	137	135-145 mEq/L
Potassium	3.3	3.5-5.0 mEq/L
ALT(SGPT)	12	7-56 U/L
AST (SGOT)	24	10-40 U/L
Total protein	8	6.0-8.3 g/dL
Albumin	2.6	3.5-5.0 g/dL
Total bilirubin	0.5	0.1-1.2 mg/dL
Bilirubin conjugated	0.1	0-0.3 mg/dL
Bilirubin unconjugated	0.4	0.2-0.8 mg/dL
Globulin (calculated parameter)	5.4	2.0-3.5 g/dL

**Table 2:** Pulmonary function test showing significant post-bronchodilator improvement in FVC and FEV1 indicative of reversible airway obstruction Pred (predicted): This refers to the predicted value of a particular pulmonary function parameter based on reference standards. M. pre (measured pre-bronchodilator): This indicates the measured value of the pulmonary function parameter before the administration of a bronchodilator. %pred (percent predicted pre-bronchodilator): This represents the percentage of the predicted value that the pre-bronchodilator measured value represents. M. post (measured post-bronchodilator): This indicates the measured value of the pulmonary function parameter after the administration of a bronchodilator. %pred (percent predicted post-bronchodilator): Similar to the pre-bronchodilator %pred, this value represents the percentage of the predicted value that the post-bronchodilator measured. %IMP (% improvement): This indicates the percentage improvement in the pulmonary function parameter after the administration of the bronchodilator. FEV1, forced expiratory volume in the first second; FVC, forced vital capacity

Parameter	Pred	M. pre	%pred	M. post	%pred	%IMP
FVC (L)	02.99 L	01.45 L	048%	02.60 L	087%	+79%
FEV_1_ (L)	02.32 L	00.61 L	026%	01.13 L	049%	+05%
FEV_1_/FVC (%)	77.59%	42.07%	054%	43.46%	056%	+03%

Treatment

Treatment of bronchiectasis was initiated in our ward once the condition was confirmed with a CT scan. We started initial intravenous doxycycline 100 mg twice daily for three days and intravenous meropenem 1 g thrice daily for four days. Additional oral azithromycin 500 mg once a day for three days was prescribed. To treat airflow blockage, this patient was prescribed inhaled bronchodilators and corticosteroids to enhance the patency of the airway. The most imperative strategy in treating bronchiectasis involves the removal of mucus. The patient was taught postural drainage, which should be done for five to 10 minutes twice a day, and vigorous breathing strategies to help remove secretions. Chest percussion, as well as the use of mechanical oscillatory devices, also promoted the mobilization and evacuation of mucus. More so, advice about avoiding tobacco and reducing biomass to prevent further lung damage. The holistic approach is done to ensure optimal management of the patient with bronchiectasis, which in turn should improve lung function and a better quality of life.

Follow-up

The patient was counseled for the Influvac Tetra vaccine, and a metered dose inhaler (MDI) of triple-drug therapy was advised, which helped the patient to relieve his symptoms. Pulse azithromycin 250 mg alternate-day therapy was given for 90 days. The patient was advised to regular follow-up.

## Discussion

The present case study discusses a male patient with a background of TB, which further developed into bronchiectasis [4]. Irreversible bronchial dilation and loss can result in cases of protracted infection with inflammation, as in most cases observed in post-infectious bronchiectasis, more commonly reported from countries with high incidences of TB [5]. The incidence of bronchiectasis appears to be notably higher in rural areas where TB is prevalent, suggesting that geographic and socio-economic factors play a critical role in disease outcomes. In such regions, limited access to healthcare services and delayed diagnosis of TB could exacerbate the progression to bronchiectasis. Consequently, early detection and management of TB in these settings are crucial in preventing the development of bronchiectasis. Factors influencing outcomes in TB patients who develop bronchiectasis include the severity and duration of the initial infection, the effectiveness of the TB treatment regimen, and the presence of other comorbid conditions. Past TB infections that were inadequately treated or managed may contribute to worse outcomes, with persistent inflammation and damage to the bronchial structures increasing susceptibility to bronchiectasis. The presence of negative TB titers in patients could indicate latency or a past infection that has been effectively treated. However, even with negative titers, residual lung damage from prior infections can still lead to chronic respiratory issues, including bronchiectasis. This underscores the importance of not only treating TB but also monitoring long-term respiratory health to reduce post-infectious complications. The patient's history of TB most probably triggered a cycle of mucociliary dysfunction, recurrent infections, and chronic inflammation in bronchiectasis [6]. Clinical manifestations include chronic coughing, production of phlegm, and recurrent respiratory infections that negatively affect quality of life [7]. Treatments for the disease are thus aimed at controlling infections, enhancing airway clearance, and reducing inflammation to prevent the progression of the illness [8]. This case also brings out the importance of integrated health approaches in the management of the complex needs of patients with co-infection of infectious and chronic respiratory conditions as a way of enhancing early detection and treatment of TB that might have otherwise reduced the risk of post-infectious bronchiectasis [9].

## Conclusions

This case of advanced bronchiectasis management was started based on investigational support from a CT scan and speaks for itself regarding integrated management for proper management. Oral azithromycin, along with supportive drugs like pantoprazole and ondansetron, as well as intravenous antibiotics like doxycycline and meropenem, act against infection and gastrointestinal side effects. Along with chest physiotherapy, bronchodilators like acebrophylline, mucolytics such as N-acetylcysteine enhance airway clearance, and protein powder and vitamins provide nutritional supplementations in the recovery phase. Such pharmacological and non-pharmacological management of bronchiectasis has indicated the fact that from a holistic point of view, bronchiectasis has to be dealt with in many ways and manner by which patient results, and quality of life can be improved.

## References

[1] King PT (2009). The pathophysiology of bronchiectasis. Int J Chron Obstruct Pulmon Dis.

[2] Kim C, Kim DG (2012). Bronchiectasis. Tuberc Respir Dis (Seoul).

[3] Sami R, Zohal M, Khanali F, Esmailzadehha N (2021). Quality of life and its determinants in patients with noncystic fibrosis bronchiectasis. J Res Med Sci.

[4] Akram A (2020). Tuberculosis-induced bronchiectasis complicated by recurrent respiratory tract infections and renal amyloidosis: a classic revisited. Cureus.

[5] Tsang KW, Bilton D (2009). Clinical challenges in managing bronchiectasis. Respirology.

[6] Shoemark A, Ozerovitch L, Wilson R (2007). Aetiology in adult patients with bronchiectasis. Respir Med.

[7] Martin MJ, Harrison TW (2015). Causes of chronic productive cough: an approach to management. Respir Med.

[8] Goetz RL, Vijaykumar K, Solomon GM (2022). Mucus clearance strategies in mechanically ventilated patients. Front Physiol.

[9] Choi H, Lee H, Ra SW (2021). Clinical characteristics of patients with post-tuberculosis bronchiectasis: findings from the KMBARC registry. J Clin Med.

